# Linking Water Quality and Quantity in Environmental Flow Assessment in Deteriorated Ecosystems: A Food Web View

**DOI:** 10.1371/journal.pone.0070537

**Published:** 2013-07-24

**Authors:** He Chen, Lekuan Ma, Wei Guo, Ying Yang, Tong Guo, Cheng Feng

**Affiliations:** 1 State Key Laboratory of Water Environment Simulation, School of Environment, Beijing Normal University, Beijing, China; 2 Chinese Academy for Environmental Planning, Beijing, China; 3 Research Center for Ecological Engineering and Nonlinear Science, North China Electric Power University, Beijing , China; 4 Biodiversity and Ecological Modelling and DCPS, Institute of Biology, Freie Universität Berlin, Berlin, Germany; University of Sydney, Australia

## Abstract

Most rivers worldwide are highly regulated by anthropogenic activities through flow regulation and water pollution. Environmental flow regulation is used to reduce the effects of anthropogenic activities on aquatic ecosystems. Formulating flow alteration–ecological response relationships is a key factor in environmental flow assessment. Traditional environmental flow models are characterized by natural relationships between flow regimes and ecosystem factors. However, food webs are often altered from natural states, which disturb environmental flow assessment in such ecosystems. In ecosystems deteriorated by heavy anthropogenic activities, the effects of environmental flow regulation on species are difficult to assess with current modeling approaches. Environmental flow management compels the development of tools that link flow regimes and food webs in an ecosystem. Food web approaches are more suitable for the task because they are more adaptive for disordered multiple species in a food web deteriorated by anthropogenic activities. This paper presents a global method of environmental flow assessment in deteriorated aquatic ecosystems. Linkages between flow regimes and food web dynamics are modeled by incorporating multiple species into an ecosystem to explore ecosystem-based environmental flow management. The approach allows scientists and water resources managers to analyze environmental flows in deteriorated ecosystems in an ecosystem-based way.

## Introduction

Ecological requirements should be addressed urgently to attain sustainability in water management and allocation [1]. Water supply planners are proactively addressing the water needs of aquatic ecosystems by reserving environmental flows for ecosystems to prevent ecological damage [2]. Many restoration projects are focused on restoring environmental flows to improve the health of the aquatic ecosystems.

Environmental ﬂow recommendations represent the flow requirements intended to keep the ecosystem health at an acceptable level [3], [4]. Environmental flows are defined as the quantity, timing, and quality of water flows required to sustain freshwater and estuarine ecosystems, as well as the human livelihood and well-being that depend on these ecosystems [5]. The concept implies that aquatic ecosystems should be in a state in which habitat quality and biotic integrity are supported. Environmental flow has become a major issue for integrated water resources management and has been advocated as a useful rehabilitation strategy for improving surface water biota [6]. Fundamentally, conservation of aquatic ecosystems is based on the implication of environmental flows, which drive aquatic ecosystems.

Many methods are used to assess environmental flows. Most current approaches rely on simple hydrological and habitat-association methods and models to predict how changes in river flow regimes affect a specific species [7]–[13]. The majority of environmental flow assessment methods can be grouped into four reasonably distinct categories: hydrological, hydraulic rating, habitat simulation, and holistic methodologies [13], [14]. These methods can reveal generalized, possibly quantitative relationships between ecological responses and specific types of flow alterations [15].

However, flow–ecological response relationships and enhanced modeling capacity to support river flow management and ecosystem conservation have yet to be elucidated [16]. Most methods such as the Instream Flow Incremental Methodology (IFIM), have been subject to criticism, including their inability to predict discharge–biomass relationships and omission of predation and/or competition as variables in assessing the dynamics of river populations and communities [4]. Despite the potential of environmental flow assessment methods to evaluate many aquatic ecosystems with minor and moderate water regime alterations, applications in deteriorated ecosystems indicate disturbing trends. Environmental flow assessment relies on the relationship between food webs and flow regimes, which can influence its application in deteriorated ecosystems. Leading approaches of environmental flow assessment in deteriorated ecosystems should be redesigned.

### Effect of Water Quantity and Quality on River Ecosystems

Water quantity, referred to as flow regime in environmental flow assessment, considers magnitude, frequency, duration, timing, and rate of change [17]. Water quality incorporates the concentration of different constituents in the water as well as its temperature and state [18].

Many aspects of water quantity and quality are closely interlinked. Water quality can vary in importance depending on the actual water quantity and the dilution rate. Ecological components are involved in the relationship between water quality and resultant water quantity [19]. Water quality is as important as the quantity and temporal patterns of flows [16].

In a surge of developments over the past decade, scientists have recognized that water quantity is inadequate; the structure and function of a riverine ecosystem, as well as many adaptations of its biota, are jointly determined by water quantity and quality [18], [20].

Methods such as ecological reserve (the quantity and quality of water necessary to protect river ecosystems based on expert panel assessment) in South Africa aim to ensure ecological sustainability. However, most techniques in environmental flow assessment focus principally on the quantity of water required to maintain ecosystem integrity, whereas methods in water quality assessment are developed at an unequal pace [20]. The most common approach to water quality protection entails the use of protective water quality guidelines, allowing for the selection of levels of resource protection but do not link these to environmental flows [21].

### Food Web View of Deteriorated Ecosystems

Most aquatic ecosystems are highly regulated by anthropogenic activities through water resource development and water pollution, thereby influencing the availability of aquatic habitats and energy sources necessary to species [22], [23]. Food webs of these ecosystems deteriorate because of decreasing environmental flows and increasing pollution.

Hydrologic variability is one mechanism potentially linking ecosystem size to food chain length [22]. Aquatic ecosystem drying will decrease food chain length through the loss of large-bodied fishes [23]. Removing floods would cause increases of predator-resistant grazing insects, which would divert energy away from the food chain leading to predatory fish.

Ecological theories demonstrate that ecosystems are characterized by dynamic feedbacks among system components and connectivity between habitats [7]. Even if the focus of ecosystem conservation efforts is on a target species rather than whole ecosystems, a food web perspective is necessary, because the population dynamics of any species depend critically on how their resources, prey, and potential predators also respond to environmental change [12], [24].

Ecologists have long advocated shifting the focus of management away from habitat provisions for target species and toward preserving the viability of larger ecosystems [7], [25], [26]. Management of ecosystems for environmental purposes requires an understanding of environmental flows in a food web view, indicating that the ecological perspective must be shifted to consider multi-species interactions [27]. Assessment of environmental flows from a multi-species perspective requires food web dynamic models that can simulate a variety of ecological processes. Process-oriented ecological models, which consider dynamics across scales and levels of biological organization, have the potential to characterize such dynamics and are better suited to guide environmental flow management [7], [28].

We propose improvements to the methods of environmental flow assessment. We outline the recognized problems present in current environmental flow assessment techniques in deteriorated aquatic ecosystems, and describe a food web view to deal with problems in environmental flow assessment, with the role of water quantity and quality considered.

## Methods

### Study Area

The Baiyangdian wetland in North China is highly regulated by anthropogenic activities through reservoir and water pollution from Baoding, a large city. Food webs in the Baiyangdian wetland have deteriorated because of decreasing environmental flows and increasing pollution. Management of wetland for environmental purposes requires an understanding of environmental flows, which compels basic understanding of relationships between stream flow and biotic response.

**Figure 1:**
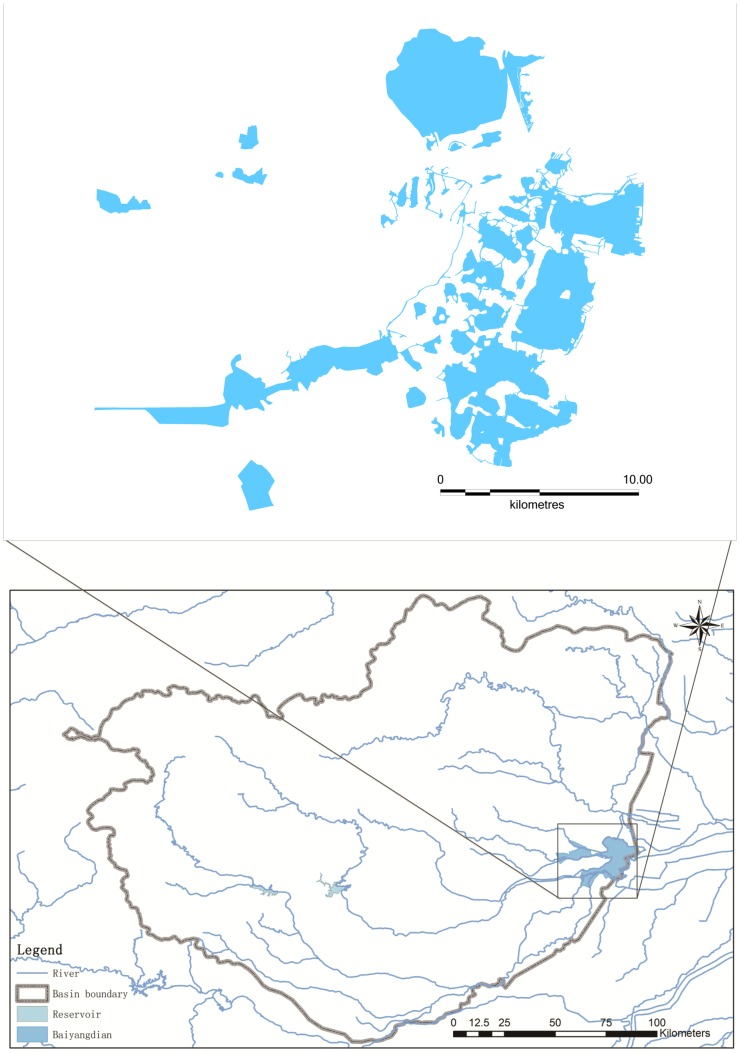
The Baiyangdian wetland and basin. The Baiyangdian wetland is the largest semi-closed wetland in the Hai River basin. Nine Rivers, with a catchment area of 31,200 km^2^, joins the Baiyangdian wetland and then flows into the Bohai Gulf.

The Baiyangdian wetland (Figure 1) is located between 115°45′E to 116°06′E longitude and 38°43′N to 39°02′N latitude and covering an area of 366 km^2^. The area is the largest semi-closed wetland in the Hai River basin. Nine upstream rivers, with a catchment area of 31,200 km^2^, join the Baiyangdian wetland and then flow into the Bohai Gulf. The elevations of the wetland range from 5.2 m to 7.8 m. In the upstream river basin, the average annual precipitation is 590 mm, and the average annual runoff is 3.10 billion m^3^. The Baiyangdian wetland supports biodiversity of regional and global significance within the Hai River Basin. The open water and aquatic beds of the wetland act as spawning grounds and feeding habitats for various fishes and other animal species, including 192 bird species.

In the past 60 years, the ecological functions of the basin and the wetland have declined with adverse impacts. The decreasing trend of precipitation can be observed in the Baiyangdian wetland in the recent 50 years. The average annual precipitation in the 1980s and 2000s had reduced by 70 mm and 143 mm respectively in comparison with that in the 1960s.

**Figure 2:**
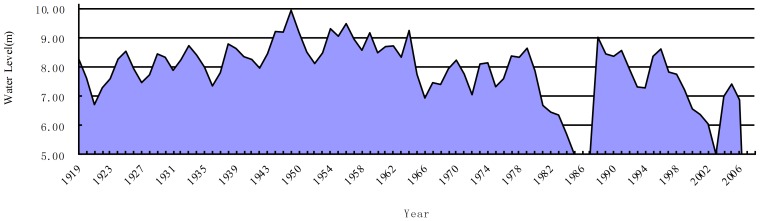
Water levels from 1919 to 2009 in the Baiyangdian wetland. The water level varied in the past 30 years. The wetland dried up from 1983 to 1988 because of deceasing environmental flows.

Since the 1950s, 143 reservoirs have been constructed in the Baiyangdian wetland basin, with a storage capacity of 3.60 billion m^3^. This capacity is higher than the annual runoff, which is 3.10 billon m^3^. The Xidayang reservoir is the largest reservoir in the Baiyangdian wetland basin. The Xidayang reservoir with a storage capacity of 1.26 billion m^3^ is located in an upstream river, Tanghe, and has a drainage area of 4,420 km^2^. The Biayangdian wetland has relied on water regulation using upstream reservoirs since 1997. Construction of large reservoirs in upstream rivers has led to a dramatic reduction in water inflows into the wetland. The average annual inflow of the Baiyangdian wetland was 2.40 billion m^3^ in the 1950s, decreasing to 1.73 billion m^3^ in the 1960s and then to 0.24 billion m^3^ in the 1980s. The wetland dried up from 1983 to 1988. Decreasing environmental flows have become more acute, especially during dry years, thus contributing to higher incidence of low water levels in the wetland (Figure 2). The size of the wetland has decreased by almost half because of controlled water flows, continuous droughts, and soil erosion. Moreover, rising human population, expanded agricultural and industrial activities, and limited solid and wastewater disposal measures in the upstream and within the wetland area have transformed the wetland into a major depository of wastewater discharges, pollutant substances, and sediment.

### Data Collection

Time series data of water level, water quality, and fishery information were collected from statistics and literature from 1919 to 2009. Biological data were obtained from comprehensive investigations covering species at different trophic levels, including the Animal Research Institute of Chinese Academy of Sciences in 1958, the Department of Biology of Hebei University in 1975, the Hebei Fisheries School in 1980, and the Baoding Institute of Environmental Sciences in 1993.

### Field Sampling

Field sampling was performed in July, October, and December 2009 and March 2010. We selected 14 sampling sites, each indicating one main separate water area in the Baiyangdian wetland. The water depth in most parts of the wetland is about 1 m. We only sampled the water at a depth of 0.5 m below the surface. These parameters include conductivity, temperature, dissolved oxygen, pH, turbidity, total nitrogen, total phosphorus, ammonia nitrogen, total organic carbon, chemical oxygen demand, chlorophyll, and blue-green algae. Conductivity (µS/cm), temperature (°C), pH, dissolved oxygen (DO) (mg/L) were measured in field by probes of water quality analyzer. Other parameters were measured in Analytical and Testing Center of Beijing Normal University, in which total nitrogen (TN) (mg/L) and total phosphorus (TP) (mg/L) were measured by elemental analyzer, and ammonia nitrogen (NH_3_-N) (mg/L), nitrate nitrogen (NO_3_-N) (mg/L), nitrite nitrogen (NO_2_-N) (mg/L) were detected by ion chromatography. Biological indices including biomass (g/m^2^) of phytoplankton (*Synedra* sp., *Merismopedia glauca, Crptomonas ovata*), zooplankton (protozoa, rotifer, cladocera and copepod), zoobenthos (mollusca, oligochaeta, aquatic insects), and macrophytes (*Phragmites australis, Typha.angustifoliaP. m aackianum, Hydrilla. Verticillata, Salvinia.natans, Ceratophyluum. demersum*) were measured according to standard methods [29]. The specific sampling and measuring methods were illustrated as follows: for phytoplankton, 0.5L water from 0.5 m below water surface was collected. Number and taxa were analyzed under microscope. For small zooplankton (protozoa and rotifer) 1L water was collected and for large zooplankton (cladocera and copepod) 10L water was collected after filtering by 25# plankton net. Small and large zooplankton was counted under microscope. Biomass of zooplankton was calculated according to average cell size of each taxon. For zoobenthos, Peterson dredge (1/16 m^2^) was used to collect twin parallel sediment samples in each site, and all zoobenthos in the sediment were identified and weighed using microscope and electronic balance. In order to get the biomass of macorphytes, 1 m×1 m quadrats were set in each site with three parallel samples. We identified and weighed the whole macorphytes in each quadrat using electronic balance. The biomass of all fishes (*Channidae, Cyprinus carpio, Carassius cuvieri, Ctenopharyn odon idellus, Hypophthalmichthys molitrix* and *Hypophthalmichthys nobilis*) was obtained from the local Fisheries Department.

Historical data rely on the collection and analysis of sediment cores. The Baiyangdian wetland suffers from a relatively severe disturbance and requires care in selecting a stable region to acquire the sediment core. The study sites were sampled in November 2009, and four sediment cores were collected from different sites of the wetland, and each core was divided into 2 cm sub-layers. A comparison experiment of sediment parameters in the parallel layer of multi-sediment cores was performed [30]. For the comparably stable region, the sedimentary condition tends to be sequential and show consistent trend. Sedimentation rate is assumed to be consistent in the entire wetland ecosystem. Ideal region for sediment core sampling rely on the anthropogenic activities. The regions with fewer disturbances were selected for acquiring historical data (i.e., southeast of the wetland). A sediment core of 80 cm was collected using the Beeker sediment sampler. The sediment core was equally separated into 40 layers and dated by ^210^Pb and ^137^Cs.

### Data Analysis

**Figure 3:**
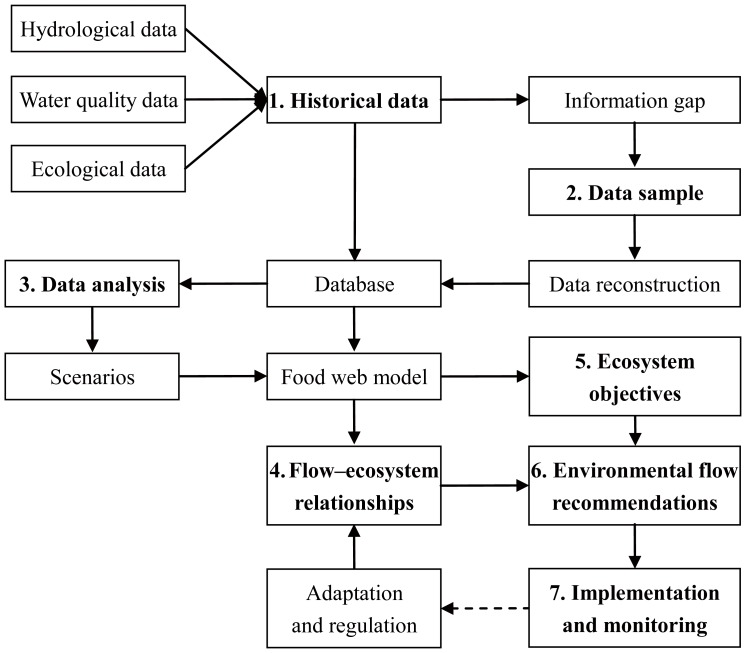
Framework for environmental flow assessment based on food web dynamics. The framework comprises seven steps: (1) collection of historical data; (2) data sampling; (3) data analysis; (4) flow alteration–ecological response relationships; (5) protection objectives; (6) flow recommendations; and (7) implementation.

We designed a process for developing environmental flow assessment based on food web dynamics to model the influence of water quantity and quality on the structure and function of the ecosystem. Our approach comprises seven steps (Figure 3): (1) collection of historical, hydrological, and ecological data and checking for key information gaps; (2) data sampling to cope with information gaps; (3) data analysis to identify hydrological, ecological, and water quality characteristics; (4) selection of an appropriate food web model to simulate the ecosystem dynamics and formulate flow alteration–ecological response relationships; (5) development of structural and functional objectives of an ecosystem; (6) initial flow recommendations; and (7) implementation of flow recommendations, monitoring of system response, and adaptation of flow recommendations.

Relationships between flow alteration and ecological characteristics are based on paired stream flow and ecological data throughout the region of interest [15]. Environmental flow assessment must incorporate an essential appreciation of flow patterns and calibrate flow standards with ecological data and other available information [14]. However, data collection may be restrained because of key information gaps. Additional data must be sampled to increase model resolution. The collected and sampled data provide initial information for environmental flow assessment. Multiple regression analysis was conducted to establish the quantitative relationship between sediment diatom species and water level, using the following equation.
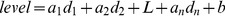
(1)where 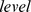 is the water level, 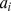 is the coefficient of diatom 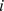, 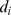 is the percentage of diatom 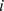, 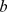 is the constant.

Environmental flow assessment requires an understanding of direct and indirect interactions between flow regimes and ecosystems. Aquatic ecosystems are highly dynamic and respond to changes in water quantity and quality in a complex manner. More efficient assessment tools are needed to describe ecological responses using coupled data sets and coupled analyses to elucidate fundamental mechanisms [31].

The food web structure is simulated using the Ecopath [32]. The Ecopath can be used to analyze the energy flow between species or groups of species in an aquatic ecosystem according to biomass estimates and food consumption relationships. The Ecopath consists of a set of linear equations representing each functional group in an ecosystem; it describes the balance between biomass gains through production and losses involved in predation, fishing, and other exports. Ecopath creates a static mass-balanced snapshot of the resources in a given ecosystem and their interactions [32]. The core components of Ecopath consist of two master equations, one for the production fate and the other for the metabolism of each group. For each living group in the model, the ecosystem is assumed to be in mass balance, which indicates that input equals output without biomass accumulation. Since Ecopath only can give an overall view of static food web structure, a dynamic model was also required to detect how different species response to water quantity and water quality. In this study, Aquatox, which released by the United States Environmental Protection Agency in 2005 was adopted to solve this problem. Aquatox has been widely used to simulate the transfer of material, energy, and chemicals in ecosystems such as wetlands, lakes, rivers, and estuaries [33], [34], [35]. In Aquatox, The driving variables include inflow, temperature, light, wind, and nutrient loadings, which can force response of abiotic and biotic state variables. This model utilizes differential equations to represent the change of state variables and uses five different libraries to save animal, plant, chemical, site and mineralization parameters respectively. The model also provides Latin hypercube uncertainty analysis, nominal range sensitivity analysis, and time-varying process rates and limitations to photosynthesis for detailed analyses [33], [36]. Hence, Aquatox model has the potential to establish links among water quantity, water quality, and biological response.

Biodiversity, integrity, and sustainability can provide an effective combination of goals that address multiple dimensions of ecosystem structure, process, and evolution, which can be met only by ecosystem-based environmental flow management [37]. Aquatic ecosystems are created, maintained, and influenced by various flow events ranging from extreme low flows to infrequent high flows. Therefore, an ecologically functional ecosystem should retain a flow regime with sufficient variability to encompass flow events that support important ecosystem processes [38], [39], [40], [41].

Concept of ecosystem maturity represented by Odum is one of the most commonly used methods to assess the status of the whole ecosystem [42]. In this research, the indices which can be quantitatively determined by Ecopath were adopted as structure and functional objectives for ecological status evaluation. TPP/TR is an important ratio describing the degree of ecosystem maturity. In mature systems, fixed energy is approximately balanced by consumption cost; thus, the ratio approaches 1 [43]. Total primary production/total biomass can reflect the succession status of the ecosystem and can take any positive value, which should be higher in a healthier ecosystem. The cycling index is the proposition of a system flow that can recycle, which should be greater than 0.5 in a mature system [44]. Path length refers to the average number of function group input or output. As the system becomes more mature, flow, recycle diversity, and length increase. The connectance index (CI) can reflect the complexity of connections in an ecosystem. The CI is high if a different function group has a complex connection in mature ecosystems. System omnivory index (SOI) is the weighted value of the overall food intake of consumers in an ecosystem. The SOI can be used for meaningful intersystem comparisons with the CI, which is strongly dependent on the level of taxonomic detail used to represent prey groups [44].

Eco-exergy equals the energy needed to decompose the system completely. Eco-exergy is a good indicator for how developed an ecosystem is and how difficult it will be to destroy it. Eco-exergy is a measure of the buffer capacity, the resistance and the inverse sensitivity of the system [45], [46].
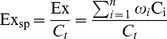
(2)where 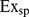
(kJ/g) is the special eco-exergy, 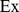
(kJ/m^3^) is the eco-exergy, 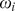 is the weight factor of specie 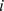, 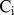 (g/m^3^) is the biomass of specie 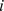, 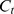 (g/m^3^) is the biomass of all species.

Implementing flow recommendations can improve the scientific understanding of flow conditions necessary to affect desired ecological changes or processes [2]. A multi-object reservoir operation model was developed, in which the environmental flow recommendations in step six were treated as limited conditions [47]. The total object function is the maximum of social and ecological benefits:
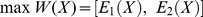
(3)

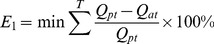
(4)

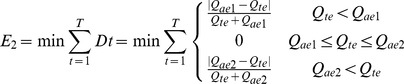
(5)where 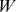 is the synthetic benefit, 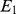 is the social benefit, 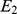 is the ecological benefit, 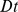 (0–1) is the distance between actual environmental flow and target environmental flow,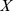 is the decision vector, 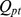 is the water requirement in time 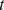, 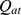 is the actual water supply in time 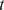, 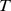 is the total operation time, 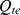 is the actual environmental flow, 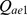 is the minimum environmental flow, and 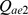 is the maximum environmental flow.

## Results and Discussion

### Historical Water Level Inference Using Sedimentary Layers

**Figure 4:**
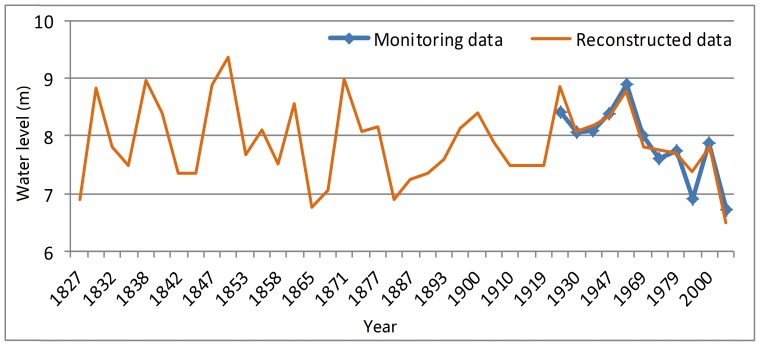
Water level in the Baiyangdian wetland from 1827 to 2006. The water level in the past 200 years reached its peak in the mid-19th century, and fluctuated fiercely between the 1820s and the 1870s and in the 20th century.

Assemblages of diatom and plant pollen in each sedimentary layer were identified and used to reconstruct historical species. Six denotable diatom species were selected to reconstruct historical water levels: *Eunotia pectinalis, Fragilaria brecistriata, Cymbella cistula, Cyclotella meneghiniana, Nitzschia palea,* and *Gomphonema gracile*. As shown in Equation 1, multiple regression analysis was conducted to establish the quantitative relationship between the plant species and the environmental variables. Correlation test indicates that each selected specie is dependent on water level based on the assumption that diatom species are inter-independent. Water levels since 1827 were reconstructed and validated using historical monitoring data since 1919 (Figure 4). Multiple regression analysis usually calls for statistical parameters to reflect the fitness effect. In this simulation, the coefficient *R^2^* is 0.982, and the *p* value is 0.018 (i.e., less than 0.05), indicating a dependent relationship between the reconstructed water level and the selected species combination. The water level in the past 200 years reached its peak in the mid-19th century. Owing to the extreme events such as droughts and floods, the water level fluctuated fiercely between the 1820s and the 1870s. Two strong fluctuations were also observed in the 20th century.

### Historical Water Level Analysis

**Figure 5:**
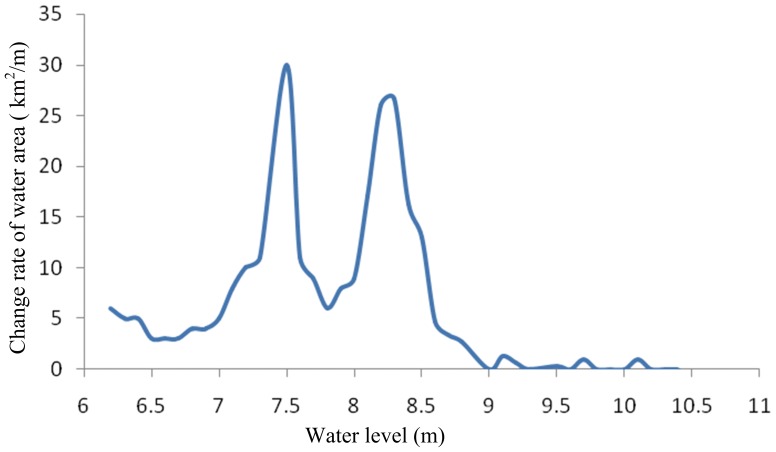
Relationship between the rate of change in water area and the water level [47]. Change rate of water level is the change of water area (km^2^) when the water level increases by 1 m. The key water levels of the Baiyangdian wetland are 7.5 and 8.3 m.

The elevation of Baiyangdian Lake ranges from 5.2 m in the east part to 6.5 m in the west part. Part of the Baiyangdian Lake will dry up when the water level is below 6.5 m. The water level of 6.5 m is fundamental important for ecological functions, above which the Baiyangdian wetland can avoid drying up. The water area of the wetland is decided by its hydraulic and topographical condition. There is a close relationship between ecosystem health and water area. According to the curve of water level and water area, the peak change rate can be calculated, which indicates the key water level for the change of water area. Based on the inferred historical water level using sedimentary layers, the relationship between the water level and the rate of change in water area is shown in Figure 5. As can be seen from the inflection points in Figure 5, the key water levels of the Baiyangdian wetland are 7.5 and 8.3 m, which should be carefully considered in environmental flow assessment.

### Flow Alteration–ecological Response Relationships

Due to lack of continuous ecological data, scattered ecological data during the past decades was collected, and four static Ecopath models were constructed based on the acceptable ecological data. Considering the comparability of model results during different period, four models were constructed following the same principle for 1958, 1980, 1993, and 2009. Each Ecopath model contained 13 function groups, which include carnivorous fish, large omnivore fish, small omnivore fish, herbivorous fishes, filter predatory fish, fingerling, mollusca, microzoobenthos, large zooplankton, microzooplankon, phytoplankton, submerged macrophyte and detritus.

**Figure 6:**
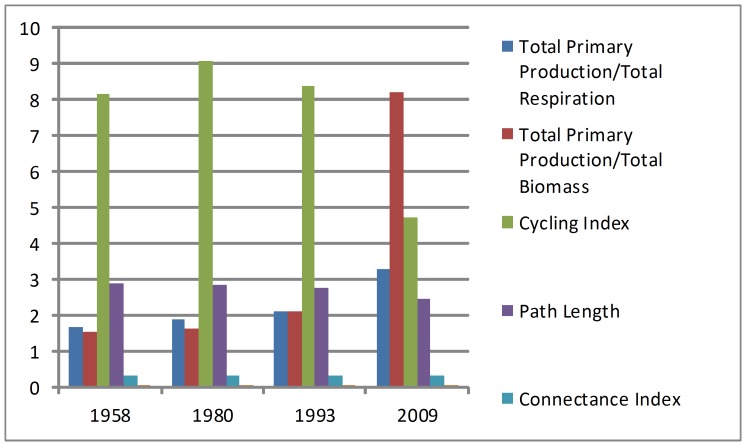
Food web structure in 1958, 1980, 1993, and 2009. Several parameters on food web structure changed sharply from 1958 to 2009. Total primary production and total respiration (TPP/TR) increased by 96.60% in the past 50 years, whereas the cycling index decreased by 41.96%.

Total primary production and total respiration (TPP/TR) increased by 96.60% in the past 50 years, whereas the cycling index decreased by 41.96% (Figure 6). Changes in other indexes were contrary to the natural processes of ecosystem development, indicating weakened interactions between organisms and further degrading system maturity.

**Figure 7:**
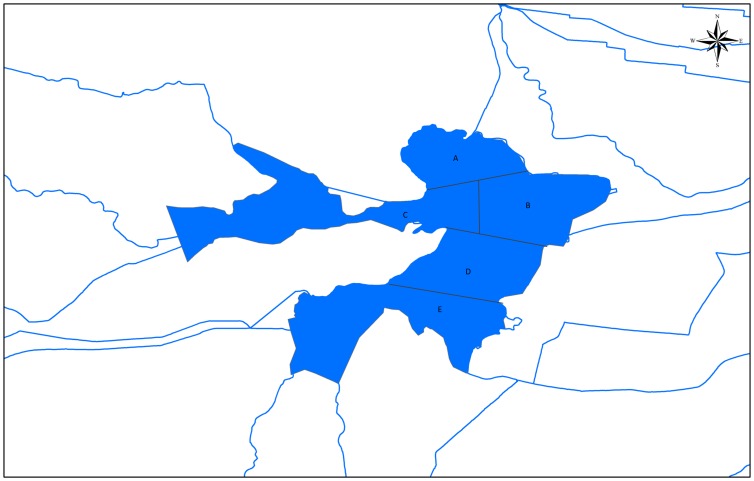
Five divided regions of the Baiyangdian wetland [32]. The Baiyangdian wetland was divided into 5 sub regions using hierarchical cluster analysis.

In this study, we determine whether the effects of environmental flows can also be incorporated into Aquatox. Only keystone species that identified by Ecosim were considered in this model because it is impossible to contain all species at different trophic level in a dynamic ecological model. Aquatox was applied to analyze the response of keystone species under different water levels and water quality conditions in the Baiyangdian wetland by using sample data, fishery statistics, and literature data [33]. We selected 14 sampling sites, each indicating one main water area, in the Baiyangdian wetland. The Baiyangdian wetland was divided into 5 subareas using hierarchical cluster analysis based on different biological communities and water quality parameters (Figure 7). 22 driving variables and state variables were considered. The abiotic variables include NH_3_-N, NO_3_-N, PO_4_^3^-, BOD_5_, DO, TSS, COD, PH, water temperature, wind velocity, light intensity, water inflow and water quantity. Diatom, green algae, blue-green algae, copepods, rotatorias, mollusks and, chironomus were selected as the biota state variables. We set the simulation time period from August 3, 2009 to September 1, 2010, and the relative error was set to 0.001. Monthly water quality data from 2009 to 2010 (derived from Baoding Environmental Science Research Institute) and the biomass data of phytoplankton, zooplankton, and zoobenthos (directly obtained from field sampling in 14 sites during four seasons from 2009 to 2010) were used for initial input and model calibration.

**Figure 8:**
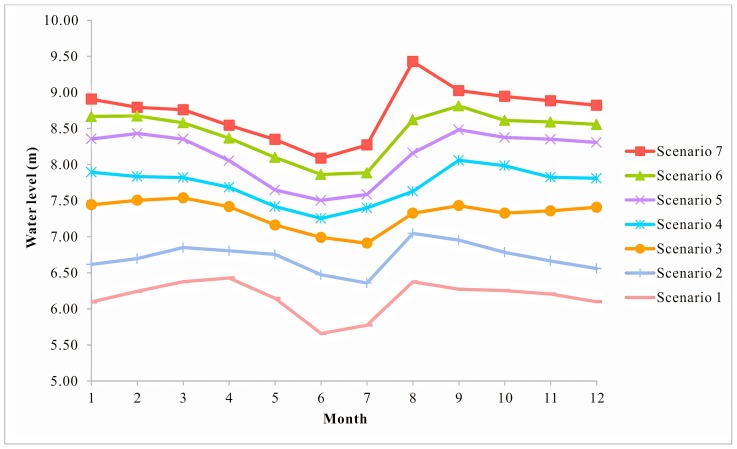
Water level scenarios. Seven water level scenarios were set, which correspond to water level frequency P = 87.5%, P = 75%, P = 62.5%, P = 50%, P = 37.5%, P = 25%, P = 12.5%.

In order to examine the response of species to environmental flows with different water quantity and quality, a total of 21 scenarios were designed. Firstly, the average monthly water level series from 1959 to 2008 were sequenced in descending order as: *x*_1_, *x*_2_, …, *x_m_*, …, *x_n_*. The experience frequency greater than *x_m_* were calculated. Then the average monthly water levels which correspond to characteristic frequency P = 12.5%, P = 25%, P = 37.5%, P = 50%, P = 62.5%, P = 75%, P = 87.5% were calculated and discretized as seven water level situations (Figure 8, Table 1). Finally, utilizing a combination of surface water quality standards class III, IV and V [48], 21 scenarios were set to analyze the synergistic effect of water quality and quantity on each trophic level organism (Table 1).

**Table 1:** Water level and simulation scenarios.

Water Level scenarios	Simulations scenarios	Water level (Frequency)	Water quality				
			Class	TN (mg/L)	NH_3_-N (mg/L)	TP (mg/L)	BOD_5_ (mg/L)
1	1	87.50%	III	1	1	0.05	4
	2	87.50%	IV	1.5	1.5	0.1	6
	3	87.50%	V	2	2	0.2	10
2	4	75%	III	1	1	0.05	4
	5	75%	IV	1.5	1.5	0.1	6
	6	75%	V	2	2	0.2	10
3	7	62.50%	III	1	1	0.05	4
	8	62.50%	IV	1.5	1.5	0.1	6
	9	62.50%	V	2	2	0.2	10
4	10	50%	III	1	1	0.05	4
	11	50%	IV	1.5	1.5	0.1	6
	12	50%	V	2	2	0.2	10
5	13	37.50%	III	1	1	0.05	4
	14	37.50%	IV	1.5	1.5	0.1	6
	15	37.50%	V	2	2	0.2	10
6	16	25%	III	1	1	0.05	4
	17	25%	IV	1.5	1.5	0.1	6
	18	25%	V	2	2	0.2	10
7	19	12.50%	III	1	1	0.05	4
	20	12.50%	IV	1.5	1.5	0.1	6
	21	12.50%	V	2	2	0.2	10

The average monthly water levels which correspond to characteristic frequency P = 87.5%, P = 75%, P = 62.5%, P = 50%, P = 37.5%, P = 25%, P = 12.5%, were calculated and discretized as 7 water level scenarios. Then, utilizing a combination of surface water quality standards class III, IV and V [48], 21 scenarios were set.

### Structural and Functional Objectives

Ecotrophic efficiency is the proportion of the production that is used in the system, and the values should be between 0 and 1(inclusive). P/Q expresses the ratio between production (P) and consumption (Q), and the value should between 0.1 and 0.3 for most groups. The pedigree routine within Ecopath software allows marking the data origin for each type of input parameters, and can provide a basis for the computation of an overall index of model “quality”. The pedigree indices of the four models were 0.584, 0.639, 0.588 and 0.582 respectively. Compared with those of 50 other previously constructed models, for which pedigree values ranged between 0.164 and 0.676 [39], the data quality in our study was available.

### Flow Recommendations

**Figure 9:**
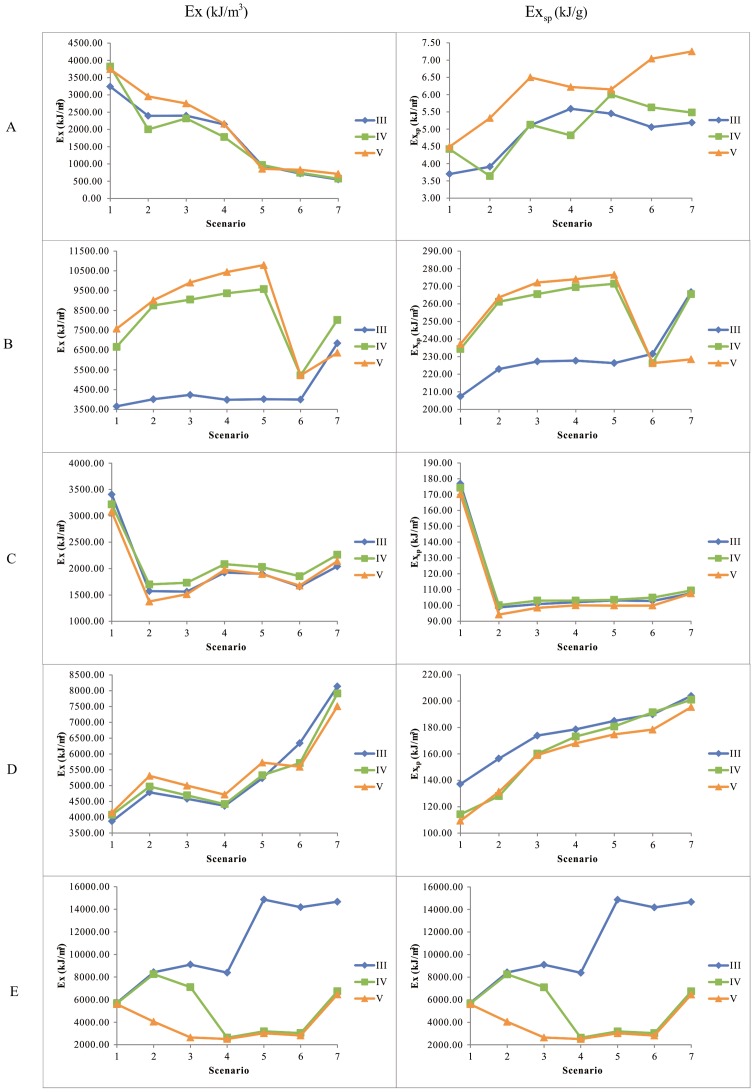
Eco-exergy and special eco-exergy in five regions. Eco-exergy and special eco-exergy in 7 water level scenarios and 3 water quality scenarios in region A, B, C, D and E.

Biomass of zoobenthos in regions A is far less than that in other regions (Figure 9). Eco-exergy increases when water level decreases. Special eco-exergy decreases when water level decreases. Eco-exergy and special eco-exergy increase when nutrient concentration increases.

The change trends of eco-exergy and special eco-exergy in region B, D and E are similar, which are positively correlated with water level (Figure 9). However, eco-exergy and special eco-exergy in region B increases when water quality decrease. Water quality in region B is relatively better than other regions. Degraded water quality will increase nutrient concentration and then stimulate organisms.

Region C is disturbed most seriously (Figure 9). The eco-exergy and special eco-exergy don’t change apparently when water level changes except for scenario 1. Restoration measures, such as sediment dredging, should be taken to promote water quality.

Each region of the wetland should be managed with different measures (Figure 7, Table 2). As shown in Table 2, water quality in five regions should be maintained in different years [48], which can meet ecosystem objectives in terms of water quality.

**Table 2:** Water quality recommendations in five regions.

Region	Water quality in dry years					Water quality in normal and wet years				
	Class	TN (mg/L)	NH_3_-N (mg/L)	TP (mg/L)	BOD_5_ (mg/L)	Class	TN (mg/L)	NH_3_-N (mg/L)	TP (mg/L)	BOD_5_ (mg/L)
A	IV	1.5	1.5	0.1	6	III	1.0	0.05	4	1.0
B	IV	1.5	1.5	0.1	6	III	1.0	0.05	4	1.0
C										
D	III	1.0	0.05	4	1.0	III	1.0	0.05	4	1.0
E	IV	1.5	1.5	0.1	6	III	1.0	0.05	4	1.0

Based on surface water features and protection targets, water standards are categorized into five classes in environmental quality standard for surface water in China, two of which are as follows: Class III for centralized drinking surface water source, fish habitat and swimming, Class IV for industrial water source or entertainment [48].

**Figure 10:**
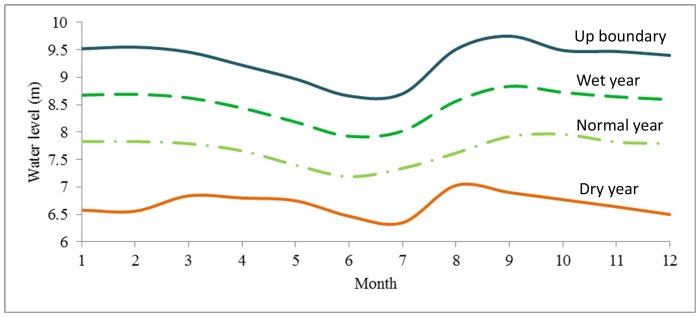
Environmental flow recommendations. Environmental flow recommendations include four components for different years: dry year, normal year, wet year and up boundary. The water level in every month should be maintained above different lines.

Recommendations were developed through flow alteration–ecological response relationships and ecosystem objectives. Limits of environmental ﬂows, referring to two boundaries, were then set according to allowable fluctuation. Recommendations were provided every day for a year (Figure 10).

### Implementation of Flow Recommendations

The Xidayang reservoirs, with a storage capacity of 1.26 billion m^3^ and a drainage area of 4,420 km^2^, is the largest reservoirs in the Baiyangdian wetland basin. Flow regulation of the Baiyangdian wetland to satisfy social and environmental benefits relies on the Xidayang reservoir.

**Figure 11:**
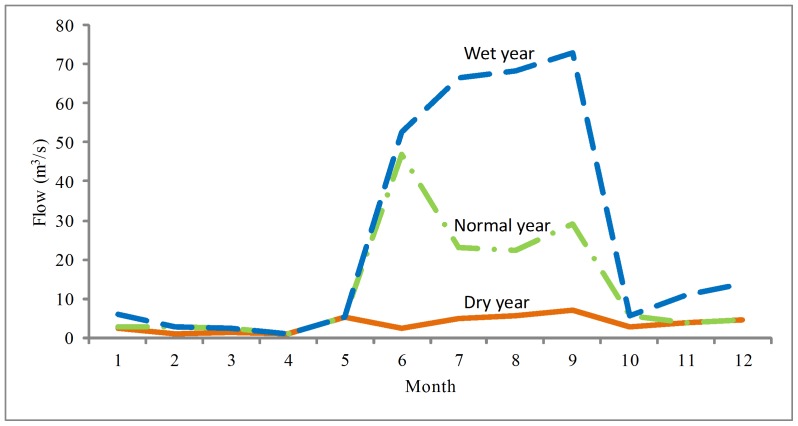
Flow recommendations of the Baiyangdian wetland in different scenarios. Inflow recommendations of the upstream rivers into the Baiyangdian wetland are calculated for dry (75%), normal (50%), and wet (25%) years.

In dry (75%) years, the social water shortage rate varies between 0.40 and 0.72 when water level is kept above the environmental flow recommendation of dry years (Figure 10). The optimal social water shortage rate is 0.40. The Xidayang reservoir should be regulated to keep the social water shortage rate above 0.40. The recommended discharge of the Xidayang reservoir in dry years is shown in Figure 11.

In normal (50%) years, the social water shortage rate varies between 0.10 and 0.72 when water level is kept above the environmental flow recommendation of normal years (Figure 10). 0.50 is a key change point of social water shortage rate, which can influence environmental flow of Baiyangdian wetland sharply. The Xidayang reservoir should be regulated to keep the social water shortage rate above 0.50. The recommended discharge of the Xidayang reservoir in normal years is shown in Figure 11.

In wet (25%) years, the social water shortage rate varies between 0 and 0.72 when water level is kept above the environmental flow recommendation of wet years (Figure 10). Social benefit and ecological benefit can both be satisfied. Social water shortage rate should be kept to 0. The recommended discharge of the Xidayang reservoir in wet years is shown in Figure 11.

As shown in Figure 10, flow recommendations of the Baiyangdian wetland are calculated for dry (75%), normal (50%), and wet (25%) years. Autumn is the wet season in the Baiyangdian wetland basin. Environmental flows should be kept at a high flow rate during the wet season. In other seasons, environmental flows can be maintained at a low limit [47]. Meanwhile, water quality should be maintained in different years in five regions, as shown in Table 2.

Two monitor stations were built to evaluate the effects of flow regulation. One station is located in the river joining the wetland. The other station is located at the center of the wetland. Flow rate (in the river), water level (in the wetland), conductivity, temperature, dissolved oxygen, pH, turbidity, total nitrogen, total phosphorus, ammonia nitrogen, total organic carbon, chemical oxygen demand, chlorophyll, and blue-green algae were monitored in two stations. Species were simultaneously sampled in September 2010 before flow regulation and November 2010 after flow regulation. Food web structure and function parameters were calculated to provide change evidence of the wetland to water resources management agency. Recommendations on adaptive flow regulation were presented to the management agency.

### Conclusions

The application of the proposed approach to environmental flow assessment in deteriorated ecosystems such as the Baiyangdian wetland revealed the major benefits of linking water quantity and quality from a food web perspective. The approach helped provide process-based flow recommendations, including water quantity and quality, by employing process-oriented ecological models that consider dynamics across scales and levels of biological organization. The proposed technique allowed for credible and practicable flow recommendations in deteriorated ecosystems.

Directly linking water quantity and quality in environmental flow assessments requires coupling of ecological dynamics with flow regimes. Many ingredients required to accomplish this task are available [7]. Water quality models enable us to simulate water pollution processes. Food web models allow us to simulate responses of ecosystem structure and function to flow regimes. However, developing required models to address ecosystem processes in environmental flow assessment remains a considerable challenge. Further research is required to link the water quantity and quality in environment flow assessment: (1) process-based ecosystem models must be improved to address ecosystem structure and function; (2) tools that link the water quantity and quality aspects of environmental flows should be fully developed to address flow regimes and ecosystem processes; and (3) long-term ecological data should be collected to verify ecosystem models.

The proposed method, which involves data sampling and model development, may seem onerous and time consuming. However, the Baiyangdian wetland application proved to be applicable. Sampling took almost one year, and model development took half a year. Environmental flows should first be assessed using traditional methods before the proposed approach can be implemented and adaptive management begins. Environmental flow recommendations can be adjusted using this approach.
